# Art and Science of Veterinary Medicine

**Published:** 1884-07

**Authors:** Robert Ward

**Affiliations:** State Veterinarian of Maryland


### Art. XVI.—ART AND SCIENCE OF VETERINARY MEDICINE.

BY DR. ROBERT WARD, F.R.C.V.S.,
*State Veterinarian of Maryland.*

Veterinary medicine was in times of old studied with some attention, for we find that Virgil, the prince of poets, and Xenophon, the great commander of armies, were both students of the art, and did not disdain to write on the subject. With the downfall of the arts and sciences in the middle ages, Veterinary Medicine sank to the bottom of the pit of darkness, and it was the last to approach the light of day. Worse indeed than Egyptian was the darkness into which it was plunged for a long course of years.

It was abandoned to the most ignorant and illiterate of men, and fell principally into the hands of those engaged in the shoeing of horses—the "Farriers"—a term signifying a worker or welder of iron, (from *ferri*, iron) and the Veterinary Art, as practiced by them, was called "Farriery"—which designation, however unmeaning, ridiculous and absurd it may be, is in some parts retained to this present day.

The practice of these rude professors was most cruel, as history tells us of the most horrid nostrums being poured down the poor brutes' throats if sick; and when kind nature herself performed a cure, despite their counteractive treatment, it was at once ascribed to the potent agency of some ridiculous compound.

Soon after human medicine had emerged from barbarism, the valuable knowledge educed from the dissection of animals, stimulated some of the more enlightened medical men in Europe to devote some of their leisure moments to the study of diseases in animals, and it was from this study that the first principles of human medicines were obtained, though at the expense of much animal suffering; for we must remember that Harvey discovered the circulation of the blood by opening the chest of a living animal, Aselli saw the lymphatics and lacteals of the mesentery in a dog; indeed all the great explorers in Medical Science made their discoveries at the expense of animal sufferings, for our benefit. The roll of names rendered illustrious at this expense is a large one, therefore the magnitude of the suffering must have been in equal ratio. Amongst those names I will mention a few that occur to me: Malpighi, Magendi, Mayo, Beaumont, Blondlot, Bernard, Lehmann, Hales, Hook, Haller and many others who labored in days gone by, and in our present day such men as Pasteur, Chauveau, Touissant and others have recently astonished the medical world with the results of their scientific experiments pertaining to the infective elements of contagious and infectious diseases among animals and mankind, all this going to show our debt to the lower animals, and our moral duty to render them some return, endowed as they are with feelings and sufferings equal to our own.

Without dwelling on the advances made from time to time, in human and animal medicine in its early history, I will pass on to that period when the Veterinary Art began to be fostered as one of the most important of the liberal sciences.

Buffon in his natural history urges most earnestly some one of the leading surgeons to take take up this neglected branch of medicine, and pleads particularly for the horse.

We find, therefore, that about this period, the early part of the eighteenth century, the first Veterinary college or school was founded at Lyons, in France, followed soon afterwards by the establishment of similar institutions in other countries, Germany and Switzerland; but it was not till nearly the close of the century that one was founded in England, and it does seem surprising that such a country, possessing the finest horses, herds and flocks in the world, should be the last to foster the science.

And I must say that it was very slow indeed in patronizing the profession at this period.

The college at St. Pancras, London, was founded through the instrumentality of an agricultural society at Odiham, in Hampshire, and it arose in this wise. A committee of Farmers, members of the society, was deputed to visit France and prepare a report on the conditiɔn of agriculture there. Whilst at Lyons, the committee visited the Veterinary school, and returned to England so deeply impressed with the great value such an institution must be to an agricultural and stock raising country, that it advised the establishment of one in London, which the society, with the assistance of the county gentry succeeded in accomplishing, and a student of the Lyons School, a M. St. Bel was engaged to superintend it. The advantages of the instruction given the practitioners from this school soon became apparent, and particularly so in the army; the government offering a commission to such members as entered the service as veterinary officers, with the rank of captain when attached. The public also came to the assistance of the profession, and speedily recognized its value, and an impetus was given it, leading well educated young men to enter as students.

The medical profession was most liberally disposed towards the young profession, the leading teachers throwing open their class-rooms to its students, Sir Chas. Bell, Sir Astley Cooper and others taking a lively interest in it.

The Royal Agricultural Society of England soon came to the aid of the profession, and established a chair of cattle pathology, which was occupied for many years by Prof. Simonds, a name well known in the agricultural world.

Scotland soon discovered its value, and in Edinburgh the well known Veterinary College was opened by the late Prof. Dick, which soon gained a high reputation in the North of England, and proved a grand success, so much so that in recent years two others have been opened in Scotland, one in Glasgow and a second in Edinburgh All of them are held in high esteem, and are prosperous—but England rests contented with the old foundation, the parent school at St. Pancras.

The profession, however, was not a chartered body till 1843, when the members were enrolled as a body corporate, under the title of the Royal College of Veterinary Surgeons of Great Britain and Ireland, but by common usage is called College of England. This college, be it noted, is distinct from the teaching schools or colleges, being the examining and diploma issuing corporation only; it has the government of the profession and framing of laws to regulate it, and advance the interest of the profession, and under its able management the art has been raised to its present high standard. To assist it, the Queen granted a supplemental charter in 1876, creating the higher degree of fellowship, thus assisting the members to prosecute their studies in practical as well as scientific research.

The Veterinary Schools or Colleges in Europe are numerous; in Germany there are several, all doing most excellent work. The one at Berlin stands I think first; the Professors are men of deep scientific research. The term of study extends over a period of four years.

In France, the school at Lyons leads for its scientific teachings with its well-known teachers, Chauveau and Colin. The school at Alford near Paris is a splendid one, and here the teaching bears more on practical surgery, most of the Army Veterinary Surgeons being trained there. The course of study extends over a period of four years, and college residence is imperative.

In England the apprenticeship system is still general, and the period ranges from one to three years, under a member of the Royal College of Veterinary Surgeons; then a collegiate course of three years qualifies for examination for the government diploma, without which it is penal to practice. For the Fellowship, five years, additional study and practical duty is needed, before being eligible for examination for the degree The leading members of the medical and veterinary professions, compose the court of examiners.

I may mention that in both Germany and France, the medical and veterinary professions are under the control of the State, and that branch of medical science which has to do with the health and well-being of the people, is termed State Medicine, which is, in other words, Sanitary Science, and the laws of Hygiene, tending to maintain the health of the people.

In England, State Medicine has not yet developed, although the foundation has been laid, and this year the Queen opens the first health exhibition.

The Veterinary profession is self supporting in England, and what it is there, the proud position it holds, is due to the *exertions* and *emulations* of its *individual members.*

I have as briefly as possible stated all this to show the position the profession holds in England, and indeed in Europe, because I regret to find that there is a somewhat general feeling in Baltimore that the veterinary profession is a degrading one. Let me say emphatically that it is not so in Europe and should not be so here.

The importance of the art and science of veterinary medicine cannot be more emphatically presented than by quoting from the "American Dairyman," of December 27th last, these statistics made by Prof. A. S. Heath:

"The United States, on January 1st, 1883, possessed two billions, two hundred and ninety millions of dollars worth of domesticated animals, representing one hundred and nineteen millions of animals, and there is only one Veterinary Surgeon to every two hundred thousand animals. The health of fifty millions of human beings depends directly and indirectly on the health of this immense live stock, which is to be guarded by the Veterinary Surgeons."

The veterinary profession is in its infancy in this country, and must be speedily matured, for, now that it has been clearly demonstrated that certain infectious and contagious diseases are communicable from animals to man, that branch of medical science, comparative pathology, must develop before long, and for its development the breach that exists between human and veterinary medicine must be bridged over, the same as it is in Europe.

Therefore it behooves communities to educate each profession, so that they may hold mutual interchange of views and ideas, looking towards the well-being of the country.

Some solid inducement must be held out to stimulate educated young men to enter the ranks of the profession. Each State should possess a veterinary institution. There is no profession at this present which can offer such a splendid field in the near future for steady, industrious young men, and I assert, there is none more ennobling.

The field of scientific research is vast and important; the study of the direct and indirect causes giving rise to disease, the nature of the contagion and its peculiarities, and the measures for arresting them, must surely prove interesting.

Let us consider the recent valuable results from the labors of Pasteur and Chauveau in this direction. The morbific germs have been recognized and removed from the blood of diseased animals. The blood has proved innocuous without the germ, and the experiments have shown that, by a modification of the virus, it may be used as a preventive medicine in arresting the ravages of direful diseases.

The recent discoveries of the savant Pasteur have produced a revolution in medical science in Europe, and this may be but the beginning of far more important discoveries.

When we remember the fact that milk from the dairy cow has brought specific germs of disease to the human family, we must recognize that, sooner or later, the public will have need of skilful medical aid for its protection.

The governments, too, will need far more assistance than they at present have at hand, should an epizootic visit us, and even now there is much activity needed, for beyond a doubt certain infectious and contagious diseases are in existence in various parts of the States, and unless the authorities exercise vigilance the diseases will, in the near future, undoubtedly give most serious trouble. The sad experience of the stock-owners of England is in history, when in 1864 the cattle plague swept off the greater part of England's best herds, and in a very great measure it was due to neglect of the veterinary profession and its counsel, which at last was accepted with happier results.

Since then the profession has been held in the highest esteem, the Queen granting the order of C. B. to its members, and the universities conferring the degree of LL.D. also, while the profession is represented on the various medical boards and assemblies.

